# Bioinformatic analysis of the expression and prognostic value of chromobox family proteins in human breast cancer

**DOI:** 10.1038/s41598-020-74792-5

**Published:** 2020-10-20

**Authors:** Xiaomin Li, Junhe Gou, Hongjiang Li, Xiaoqin Yang

**Affiliations:** 1 West China School of Medicine, West China Hospital, Sichuan University, Chengdu, People’s Republic of China; 2 Department of Pathology, West China Hospital, Sichuan University, Chengdu, People’s Republic of China; 3 Department of Breast Surgery, West China Hospital, Sichuan University, No. 37 Guoxue Alley, Wuhou District, Chengdu City, Sichuan Province People’s Republic of China

**Keywords:** Cancer, Molecular biology

## Abstract

Chromobox (CBX) family proteins control chromatin structure and gene expression. However, the functions of CBXs in cancer progression, especially breast cancer, are inadequately studied. We assessed the significance of eight CBX proteins in breast cancer. We performed immunohistochemistry and bioinformatic analysis of data from Oncomine, GEPIA Dataset, bcGenExMiner, Kaplan–Meier Plotter, and cBioPortal. We compared mRNA and protein expression levels of eight CBX proteins between breast tumor and normal tissue. The expression difference of CBX7 was the greatest, and CBX7 was downregulated in breast cancer tissues compared with normal breast tissues. The expression of CBX2 was strongly associated with tumor stage. We further analyzed the association between the eight CBX proteins and the following clinicopathological features: menopause age, estrogen receptor (ER), progesterone receptor (PR) and HER-2 receptor status, nodal status, P53 status, triple-negative status, and the Scarff–Bloom–Richardson grade (SBR) and Nottingham prognostic index (NPI). Survival analysis in the Kaplan–Meier Plotter database showed that the eight CBX proteins were significantly associated with prognosis. Moreover, CBX genes in breast cancer patients had a high net alteration frequency of 57%. There were significant co-expression correlations between the following CBX protein pairs: CBX4 positively with CBX8, CBX6 positively with CBX7, and CBX2 negatively with CBX7. We also analyzed the Gene Ontology enrichment of the CBX proteins, including biological processes, cellular components, and molecular functions. CBX 1/2/3/5/8 may be oncogenes for breast cancer, whereas CBX 6 and 7 may be tumor suppressors for breast cancer. All eight CBX proteins may be predictive for prognosis. Clinical trials are needed to confirm the significance of the eight CBX proteins in breast cancer.

## Introduction

Chromobox (CBX) family proteins are the mammalian orthologs to the heterochromatin protein 1 (HP1) and Polycomb proteins that regulate heterochromatin, gene expression, and developmental programs^1^. The HP1 orthologs CBX1, CBX3, and CBX5 share a characteristic N-terminal chromodomain, a central hinge domain, and a C-terminal chromoshadow domain^2^. The Polycomb orthologs CBX2, CBX4, CBX6, CBX7, and CBX8 have a C-terminal polycomb repressor box that serves as a canonical component in Polycomb Repressive Complex 1^3^. Dysregulation of CBX family proteins is associated with tumorigenesis of many cancers, such as breast cancer^4^, pancreatic cancer^5^, thyroid cancer^6^, colorectal cancer^7^, lung cancer^8^, and ovarian cancer^9^.

Breast cancer is the most frequent malignancy and the leading cause of cancer death among women worldwide^10^. Although many experimental and clinical investigations have been conducted for novel and less toxic treatments, and the molecular basis of the pathogenesis of breast cancer has been studied extensively, patient survival rates still need improvement^11^. Biomarkers, such as ER, PR, and HER-2, have been used widely for breast cancer prognosis and as targets of endocrine therapy or targeted therapy^12,13^.

Due to tumor heterogeneity, there is a high demand for new biomarkers to improve individualized patient treatment and prediction of outcomes. Investigators reported that eight CBX family proteins (CBX 1–8) have important functions in breast cancer^4,14–20^. CBX2 promoted breast cancer cell proliferation; its overexpression caused upregulation of genes involved in cell cycle progression, and CBX2 overexpression was associated with poor 5-year survival^14^. Upregulation of CBX4 exerted an oncogenic effect on breast cancer by the Notch1 signaling pathway^16^. However, the activities of CBXs in the development of breast cancer, as tumor promoters or suppressors, require additional research.

In this study, our goal was to predict CBX family members' functional significance in breast cancer by bioinformatics analyses of databases. We examined diverse expression patterns, clinicopathological parameters, prognostic values, including overall survival (OS), disease-free survival (DFS), post-progression survival (PPS), and distance metastasis-free survival (DMFS), genetic alterations, and gene ontology. Our findings indicated that CBXs might have complex and distinct functions in breast cancer progression.

## Materials and methods

### ONCOMINE database

ONCOMINE (www.oncomine.org)^21^ is a cancer microarray database and data-mining platform facilitating discovery from genome-wide expression analysis. Using this database, we analyzed the mRNA expression of eight CBX family proteins in breast cancer compared with normal breast tissues. We chose the Breast Cancer vs. Normal Analysis about each individual CBX protein, and the threshold included expression fold change ≥ 1.5 between cancer and normal tissues, *p* value < 0.05, and gene rank ≥ top 10%.

### GEPIA dataset

GEPIA (Gene Expression Profiling Interactive Analysis; https://gepia.cancer-pku.cn)^22^, a tool based on TCGA and GTEx data, provides RNA expression data of 9,736 tumors and 8,587 normal samples. Using this database, we performed differential expression analysis and tumor stage analysis related to each CBX protein for patients with breast cancer. In the expression analysis, the threshold included expression fold change ≥ 1.5 between cancer and normal tissues, *p* value < 0.05.

### Immunohistochemistry

We performed immunohistochemistry by using CBX2 (Abnova, monoclonal, mouse, ABN-MAB17287, 1/800, pH 6.0) and CBX7 (Invitrogen, polyclonal, rabbit, PA5-61801, 1/50, pH 7.2) antibodies in 40 pairs of paraffin-embedded invasive breast cancer issues (IBCs) and tumor-adjacent normal tissues. These sample tissues were derived from 40 patients diagnosed with primary breast cancer in West China Hospital, Sichuan University, from 2018 to 2019. The Ethics Committee of West China Hospital, Sichuan University, approved this study, and all participants signed the written informed consent.

Sections of 3 mm were cut with a microtome from the paraffin-embedded tissue blocks of IBCs and normal tissues. Then, the sections were incubated with anti-CBX2 and anti-CBX7 antibody at 4℃ overnight, covered with 3, 3-diaminobenzidine, and mounted on slides with Vectashield (Vector Laboratories). Slides were observed by light microscopy. Control experiments without primary antibody demonstrated signal specificity.

All methods were carried out in accordance with relevant guidelines and regulations. The immunohistochemistry experiment was approved by the National Key Laboratory of Biotherapy of West China Hospital, Sichuan University.

### Breast cancer gene-expression miner v4.4 (bc-GenExMiner v4.4)

The Breast Cancer Gene-Expression Miner v4.4 (https://bcgenex.centregauducheau.fr/BC-GEM/GEM-Accueil.php?js=1)^23,24^, a DNA microarray and RNA-seq database, can be used to analyze prognosis based on gene expression. Using the RNA-seq data, we evaluated the association between mRNA expression of the eight CBX family proteins and clinicopathological parameters, such as menopause age, ER, PR, HER-2, nodal status, P53 status, basal-like and TNBC status, and the Nottingham prognostic index (NPI) and Scarff–Bloom–Richardson (SBR) grading. In addition, we performed the pairwise correlation analysis of the eight CBX proteins, and we analyzed their Gene Ontology enrichment, including biological processes, cellular components, and molecular functions. Data were last updated on December 9, 2019.

### The Kaplan–Meier Plotter

The Kaplan–Meier Plotter (www.kmplot.com)^25^ is a tool to draw survival plots with gene expression data and survival information from GEO, EGA and TCGA cancer microarray datasets. We evaluated the relevance of the mRNA expression level of eight CBX proteins to the clinical outcomes (OS, RFS, PPS and DMFS) of untreated breast cancer patients. This tool automatically calculates the best cutoff value, log-rank *P* value, hazard ratio (HR), and 95% confidence intervals (CIs).

### cBioPortal

The cBio Cancer Genomics Portal (https://cbioportal.org)^26,27^ is a resource for interactive exploration of multidimensional cancer genomics datasets. We analyzed the gene alteration frequency and co-expression of eight CBX family proteins using METABRIC data from 1904 breast cancer patients^28^. The mRNA expression z-score threshold was ± 1.5 between the unaltered and altered patients.

## Results

### The mRNA and protein expression of CBXs in breast cancer

**Figure 1:**
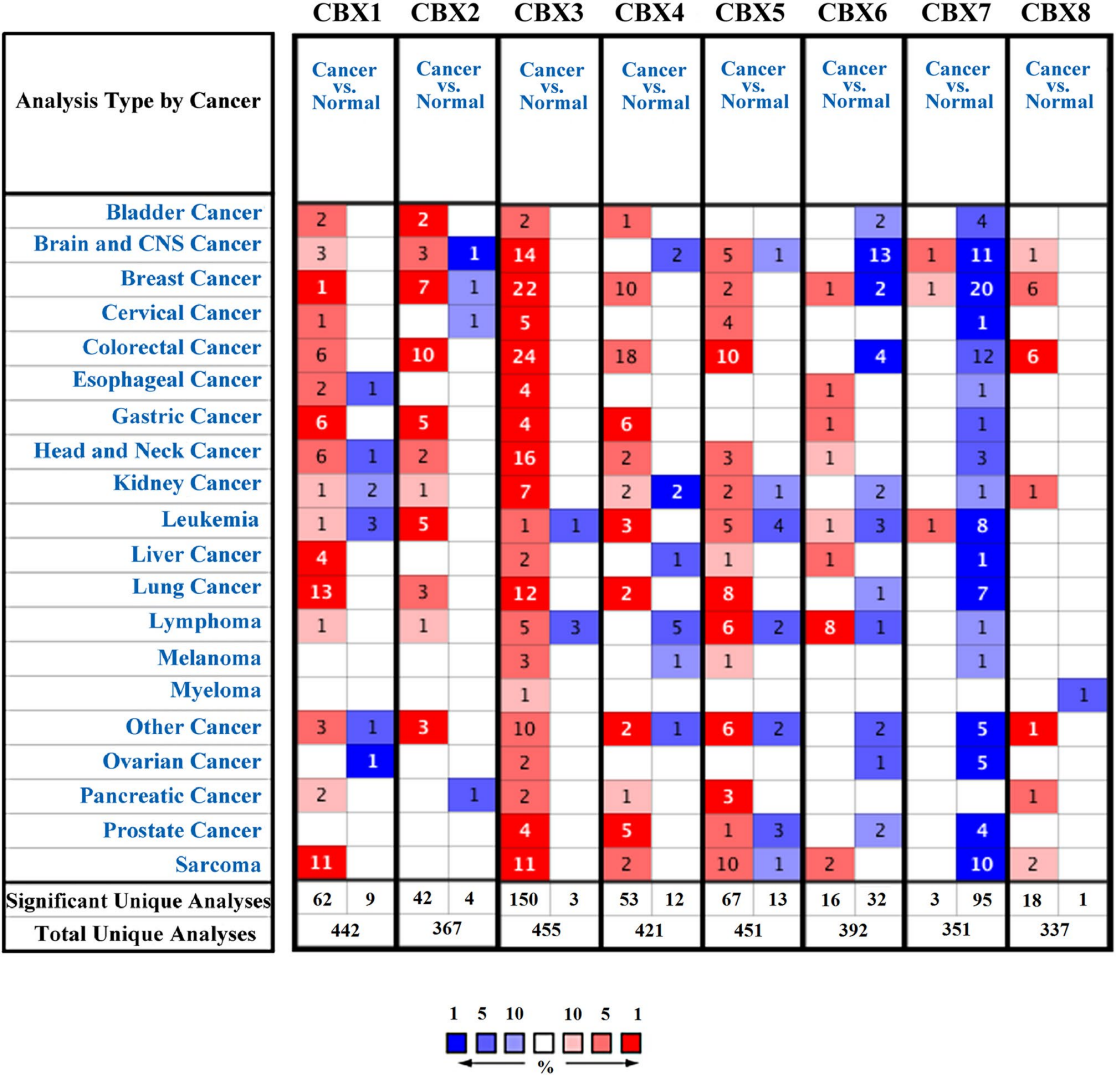
The mRNA expression of eight CBX proteins in various cancer types in Oncomine. Red: overexpression or copy gain; Blue: underexpression or copy loss. Color intensity indicates the best rank of the gene in the analyses. The number in each cell is the number of analyses that met our thresholds.

We used the Oncomine and GEPIA databases to retrieve mRNA expression levels of the eight CBX proteins in breast cancer. Oncomine analysis revealed the mRNA expression of the eight CBX proteins in 19 common types of cancer and their comparisons with normal tissues (Fig. 1). The following expression patterns were observed for breast cancer: Overexpressed, CBX1, one of 49 (1/49) analyses, CBX 2–8, 7/43, 22/53, 10/53, 2/53, 1/52, 1/41, and 6/42, respectively. Downregulated, CBX2, CBX6, and CBX7, 1/43, 2/52, and 20/42, respectively.

**Figure 2:**
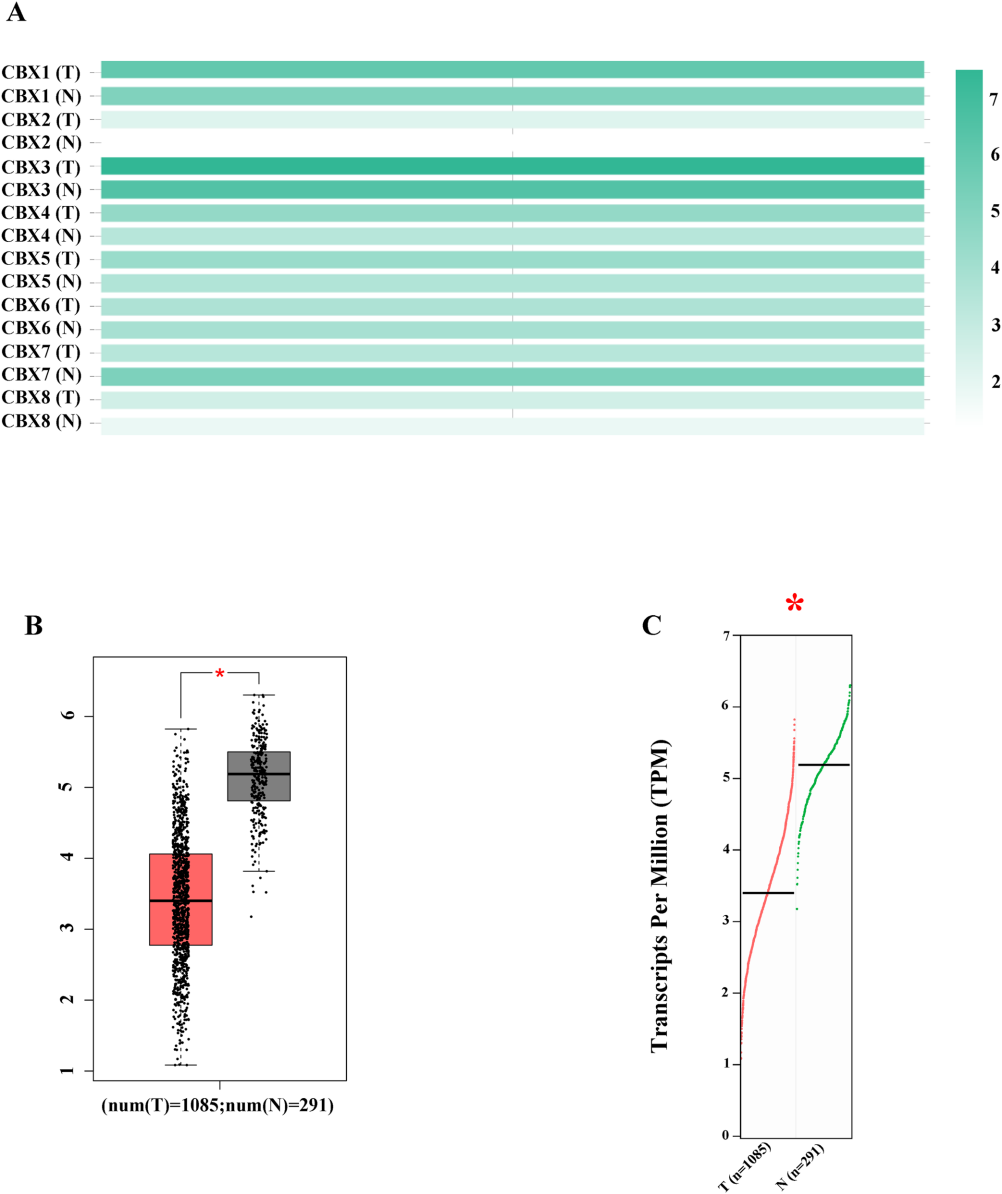
The mRNA expression of CBX proteins in breast tumors and normal tissues in GEPIA. (**A**) Eight CBX proteins. Color intensity indicates the mRNA expression of the gene in the tissue. (**B**) CBX7 mRNA expression on the box plot. (**C**) CBX7 mRNA expression profile; red: tumor tissue, Green: normal tissue; *P < 0.05 and |Log2 (fold-change)| cutoff = 1.5. We used a log scale to show mRNA expression level.

Figure 2A and Supplementary Figure 1A-B show the mRNA expression of eight CBX proteins in GEPIA. CBX7 was downregulated in tumor samples compared with the normal counterpart (*P* < 0.05; Fig. 2B,C).

**Figure 3:**
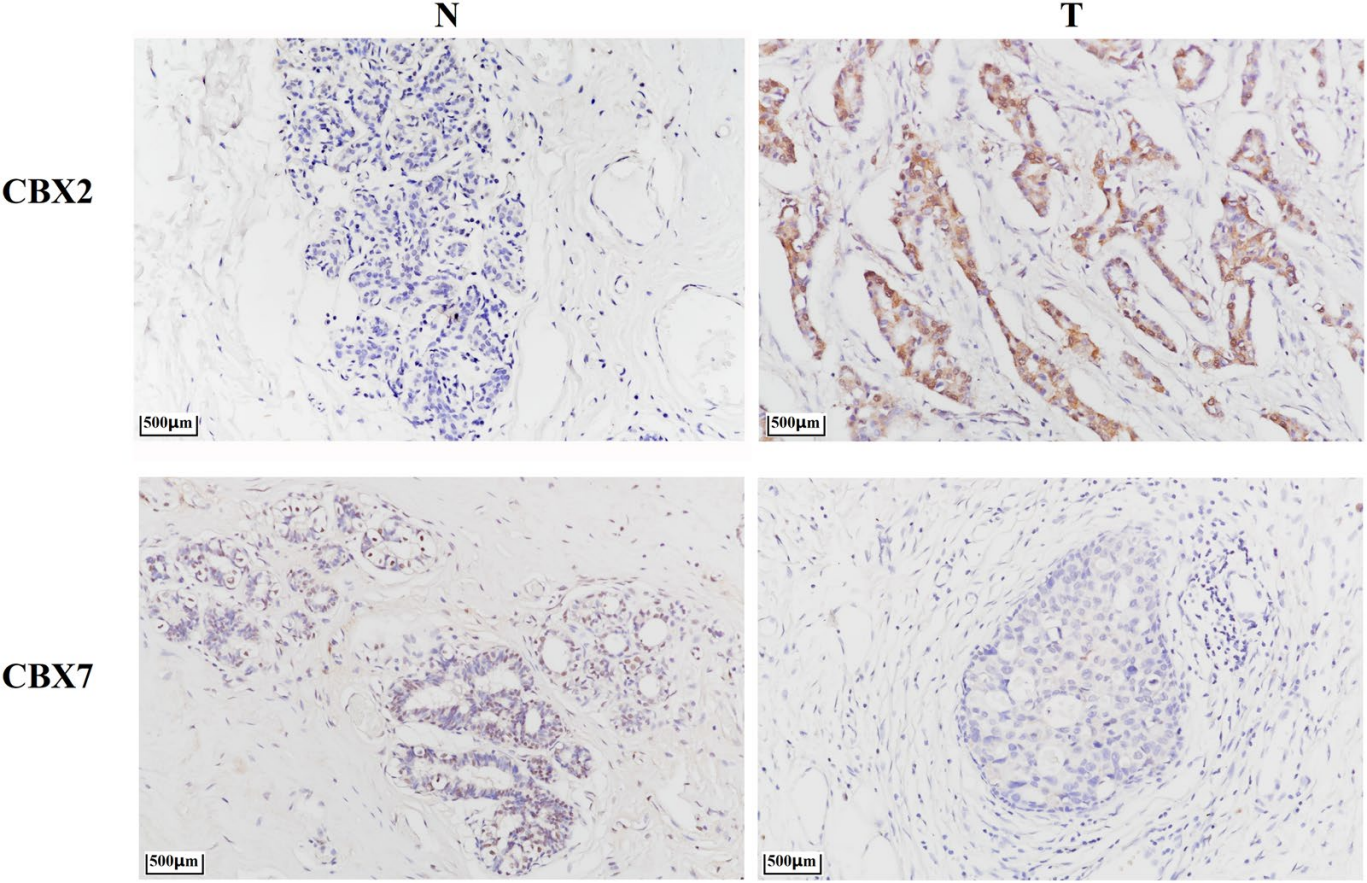
CBX2 and CBX7 protein expression in breast cancer and tumor-adjacent normal tissues. N: tumor-adjacent normal tissues; T: tumor tissues.

We performed immunohistochemistry to measure CBX2 and CBX7 protein expression (Fig. 3). We found that CBX2 protein was highly expressed in the breast cancer tissues compared with normal tissues, and expression of CBX7 protein in breast cancer tissues was lower than tumor-adjacent normal tissues.

### Associations between CBXs and the clinicopathological parameters of patients with breast cancer

**Figure 4:**
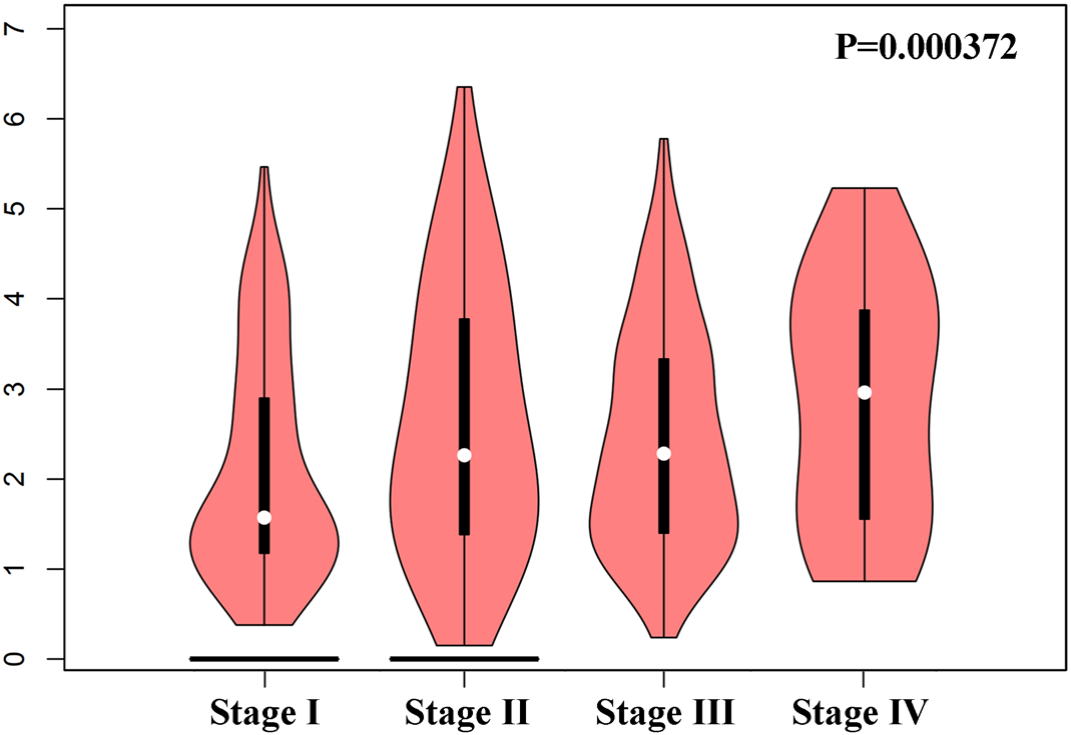
Association between CBX2 and clinical stages of breast cancer patients. The y axis: log2(TPM + 1) (TPM: transcript per million).

Table 1 shows the clinicopathological parameters and associations derived from the analysis of 4712 breast cancer patients in the TCGA and GSE81540 RNA-Seq datasets in bc-GenExMiner v4.4. High CBX2 correlated with young menopause age (*P* < 0.0001), whereas high CBX4 and CBX7 were associated with old advanced menopause age (*P* < 0.0001). CBX2 was negatively associated with ER and PR expression and positively with HER-2 expression. However, CBX 4–7 were positively associated with ER and PR expression and negatively with HER-2 expression. Patients with high CBX 1–4 were more likely to be in positive nodal status, but patients with high CBX 6–7 tended to be in negative status. Except for CBX5 and CBX8, the other six CBX proteins were correlated with P53 status. Patients with high CBX 1–3 and low CBX 4–7 were more likely to be TNBC phenotype. Concerning the prognostic factors, Scarff–Bloom–Richardson grade (SBR) and Nottingham prognostic index (NPI) in breast cancer, high CBX 1–4 and CBX8 had high SBR and NPI; by contrast, high CBX 5–7 had low SBR and NPI. CBX2 was positively associated with clinical stages of patients (Fig. 4). Stage IV patients had higher CBX2 expression compared with other stages. Supplementary Figure 2 shows the Association associations between other CBX proteins and clinical stage.

**Table 1:** Correlation between clinicopathological parameters and (A) CBX 1–4, (B) CBX 5–8.

	CBX1	CBX2
**(A)**		
Age	≤ 51 = > 51 (*P* = 0.1740)	≤ 51 > > 51 (*P* < 0.0001)
ER	+ < − (*P* = 0.0168)	+ < − (*P* < 0.0001)
PR	+ = − (*P* = 0.6980)	+ < − (*P* < 0.0001)
HER-2	+ > − (*P* = 0.0175)	+ > − (*P* < 0.0001)
Nodal status	+ > − (*P* = 0.0167)	+ > − (*P* = 0.0079)
P53 status	Wild type < mutated (*P* = 0.0007)	Wild type < mutated (*P* < 0.0001)
TNBC status	TNBC > not TNBC (*P* = 0.0341)	TNBC > not TNBC (*P* < 0.0001)
SBR	SBR1 < SBR2 < SBR3 (*P* < 0.0001)	SBR1 < SBR2 < SBR3 (*P* < 0.0001)
NPI	NPI1 < NPI2 = NPI3 (*P* < 0.0001)	NPI1 < NPI2 < NPI3 (*P* < 0.0001)

	CBX3	CBX4
Age	≤ 51 = > 51 (*P* = 0.0843)	≤ 51 < > 51 (*P* < 0.0001)
ER	+ < − (*P* = 0.0040)	+ > − (*P* < 0.0001)
PR	+ = − (*P* = 0.4357)	+ > − (*P* < 0.0001)
HER-2	+ = − (*P* = 0.1457)	+ < − (*P* = 0.0005)
Nodal status	+ > − (*P* = 0.0003)	+ > − (*P* = 0.0130)
P53 status	Wild type < mutated (*P* < 0.0001)	Wild type > mutated (*P* < 0.0001)
TNBC status	TNBC > not TNBC (*P* = 0.0115)	TNBC < not TNBC (*P* < 0.0001)
SBR	SBR1 < SBR2 < SBR3 (*P* < 0.0001)	SBR1 < SBR2 = SBR3 (*P* = 0.0083)
NPI	NPI1 < NPI2 < NPI3 (*P* < 0.0001)	NPI1 < NPI2 = NPI3 (*P* = 0.0005)

	CBX5	CBX6
**(B)**		
Age	≤ 51 = > 51 (*P* = 0.4356)	≤ 51 = > 51 (*P* = 0.9725)
ER	+ > − (*P* < 0.0001)	+ > − (*P* < 0.0001)
PR	+ > − (*P* < 0.0001)	+ > − (*P* = 0.0002)
HER-2	+ < − (*P* < 0.0001)	+ < − (*P* < 0.0001)
Nodal status	+ = − (*P* = 0.3564)	+ < − (*P* < 0.0001)
P53 status	Wild type = mutated (*P* = 0.8605)	Wild type > mutated (*P* < 0.0001)
TNBC status	TNBC < not TNBC (*P* = 0.0005)	TNBC < not TNBC (*P* = 0.0009)
SBR	SBR1 > SBR2 > SBR3 (*P* < 0.0001)	SBR1 > SBR2 > SBR3 (*P* < 0.0001)
NPI	NPI1 > NPI2 > NPI3 (*P* < 0.0001)	NPI1 > NPI2 > NPI3 (*P* < 0.0001)

	CBX7	CBX8
Age	≤ 51 < > 51 (*P* < 0.0001)	≤ 51 = > 51 (*P* = 0.4638)
ER	+ > − (*P* < 0.0001)	+ > − (*P* = 0.0083)
PR	+ > − (*P* < 0.0001)	+ = − (*P* = 0.8690)
HER-2	+ < − (*P* < 0.0001)	+ > − (*P* = 0.0144)
Nodal status	+ < − (*P* = 0.0174)	+ = − (*P* = 0.0932)
P53 status	Wild type > mutated (*P* < 0.0001)	Wild type = mutated (*P* = 0.7531)
TNBC status	TNBC < not TNBC (*P* < 0.0001)	TNBC = not TNBC (*P* = 0.6027)
SBR	SBR1 > SBR2 > SBR3 (*P* < 0.0001)	SBR1 < SBR2 < SBR3 (*P* < 0.0001)
NPI	NPI1 > NPI2 > NPI3 (*P* < 0.0001)	NPI1 < NPI2 < NPI3 (*P* < 0.0001)

*CBXs* chromobox family proteins, *ER* estrogen receptor, *PR* progesterone receptor, *HER-2* human epidermal growth factor receptor 2, *P53* a tumor suppressor gene, *TNBC* triple-negative breast cancer, *SBR* the Scarff–Bloom–Richardson (SBR) grade, *NPI* the Nottingham prognostic index.

### The prognostic value of CBXs

**Figure 5:**
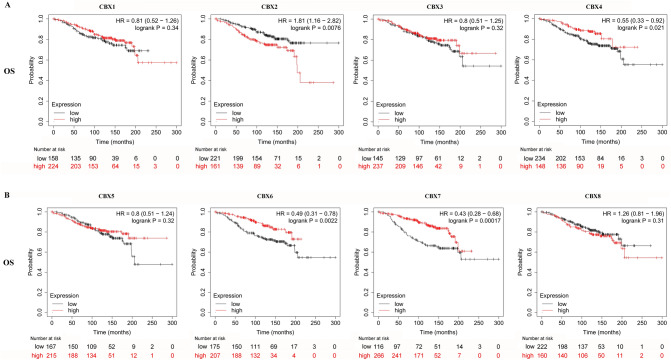
Prognostic values of CBX proteins for overall survival (OS). (**A**) CBX 1–4; (**B**) CBX 5–8.

Prognosis analysis by the Kaplan–Meier Plotter revealed that all eight CBX proteins had predictive value for the relapse-free survival of breast cancer patients (Supplementary Figure 3). Decreased CBX2 and increased CBX 4/6/7 mRNA levels were remarkably associated with longer overall survival (Fig. 5). Patients with high CBX 1/7/8 mRNA levels had longer post-progression survival than the low counterparts (Supplementary Figure 3). Moreover, decreased CBX 1/2/3/5 and increased CBX 6/7 mRNA levels were significantly correlated with longer distance metastasis-free survival (Supplementary Figure 3).

### Alterations and co-expression of CBXs

**Figure 6:**
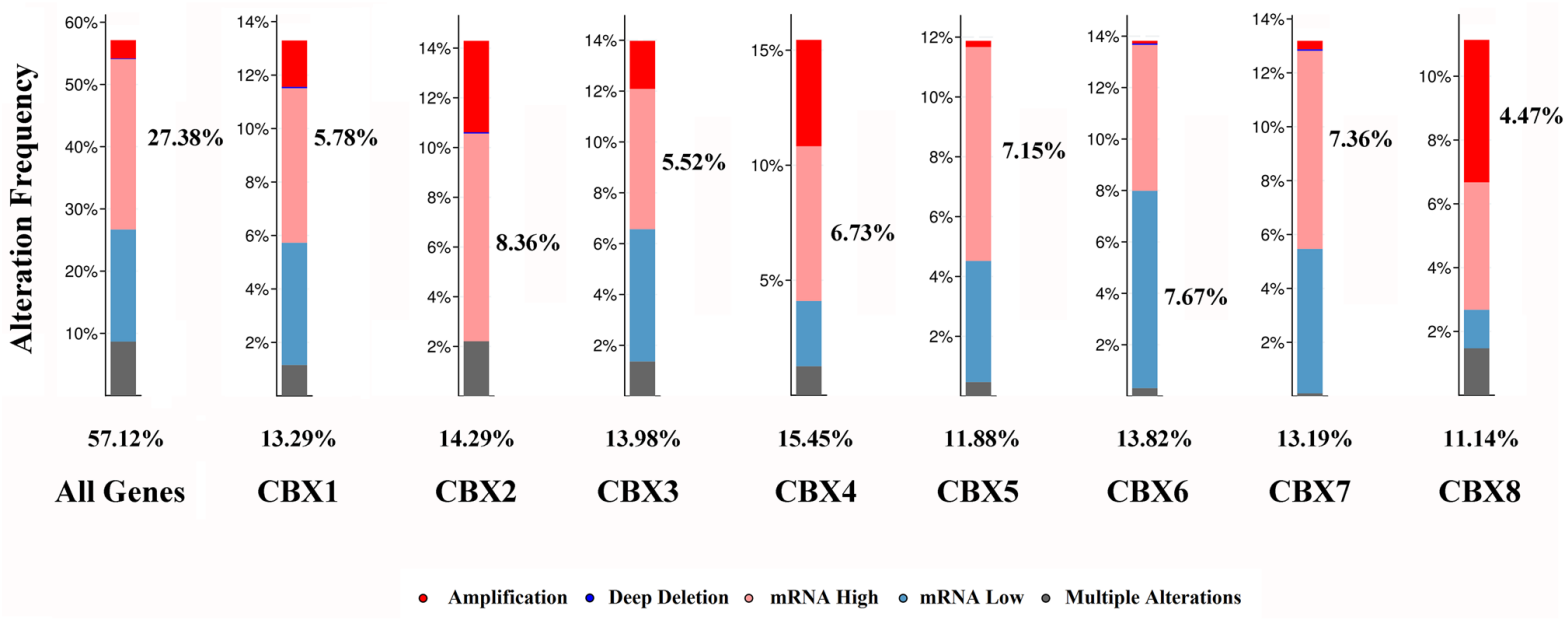
The alteration frequency and mechanisms for CBX proteins.

**Figure 7:**
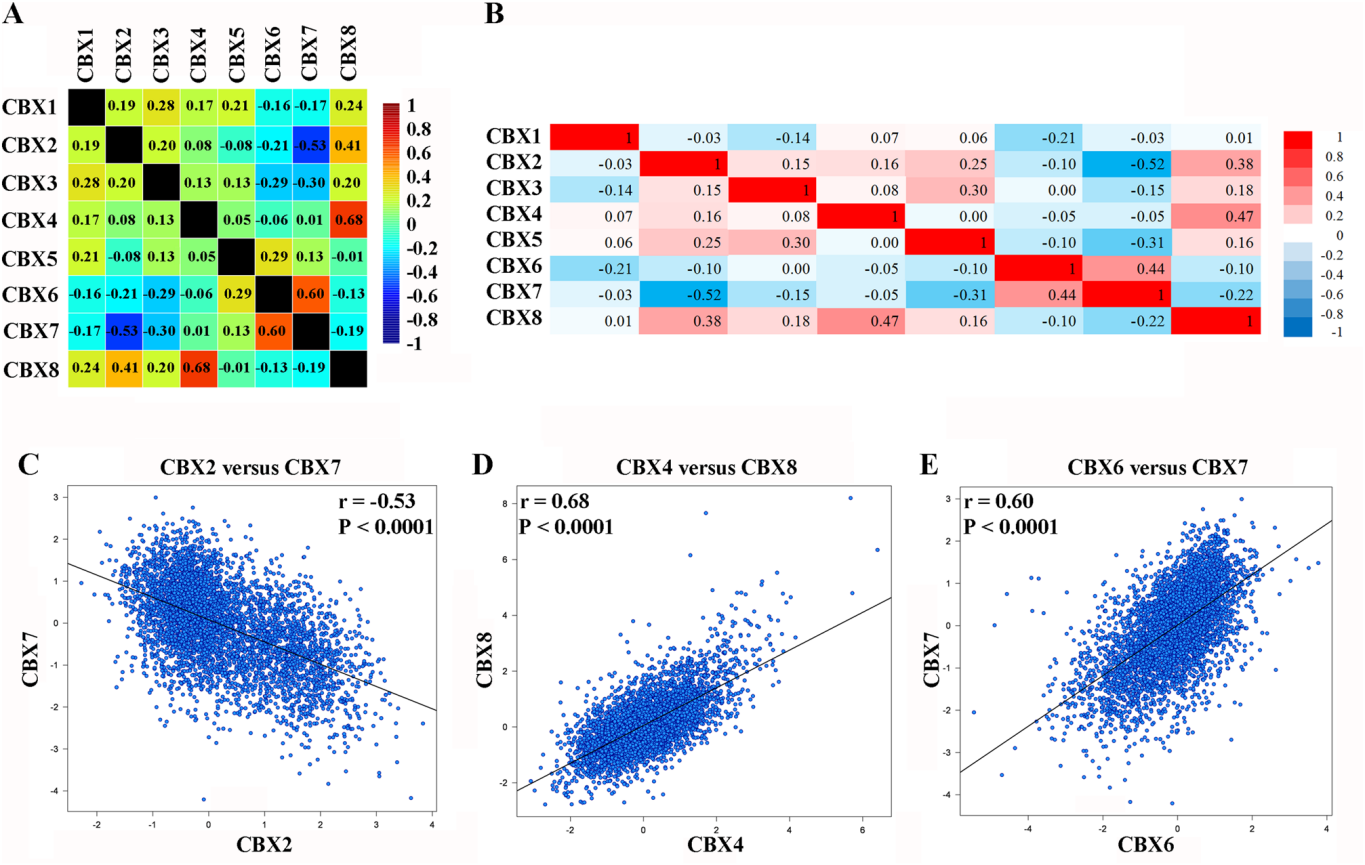
Pearson’s correlations for mRNA expression of pairwise combinations of CBX proteins in cBioPortal (**B**) and bc-GenExMiner (**A**, **C**–**E**). Tables (**A**), (**B**) include Pearson correlation coefficients, and p values of the coefficients are shown in Supplementary Table s1. The color scale interprets the correlation coefficient value. (**C**)–(**E**) show the correlation between CBX2 and CBX7, CBX4 and CBX8, and CBX6 and CBX7, respectively. *r* Pearson’s correlation coefficient value; *P*: *P* value; *No* the number of patients.

Using cBioPortal, we analyzed genetic alterations of the eight CBX proteins and found a high alteration frequency (57%) in breast cancer patients (Fig. 6). Patients who had CBX4 alteration were the most cases in eight CBX family proteins, making up 15.45% of all cases involved, and their primary alteration type was mRNA high. Besides, there were co-expression correlations between the following CBX proteins: CBX4 positively with CBX8, CBX6 positively with CBX7, and CBX2 negatively with CBX7 (Fig. 7B). The bc-GenExMiner produced similar correlations (Fig. 7A,C–E).

### CBXs gene ontology enrichment

Using bc-GenExMiner, we found the 50 (or fewer) genes most correlated to each CBX protein. Some genes were positively correlated with CBX protein, whereas some were negatively correlated with the protein. We performed gene ontology analysis of each CBX protein for biological processes (Supplementary Table 2 and Table 2; the most significant term), cellular components (Supplementary Table 3 and Table 3), and molecular functions (Supplementary Table 4 and Table 4).

**Table 2:** Biological process.

Genes	Significant terms	Description	*p* value	Associated genes
CBX1	GO:1900182	Positive regulation of protein localization to nucleus	4.45E−04	CDK5RAP3, KAT7
CBX2	GO:0045137	Development of primary sexual characteristics	1.14E−03	CBX2
CBX3	GO:1905007	Positive regulation of epithelial to mesenchymal transition involved in endocardial cushion formation	3.03E−05	ENG, TGFBR2
CBX4	GO:0042552	Myelination	6.37E−04	FAM126A, QKI
CBX5	GO:0070317	Negative regulation of G0 to G1 transition	4.45E−03	CBX5
CBX6	GO:0006325	Chromatin organization	1.69E−04	CBX6, CBX7
CBX7	GO:0051301	Cell division	7.24E−30	AURKA, BIRC5, BUB1, BUB1B, CCNA2, CCNB1, CCNE2, CDC20, CDCA2, CDCA3, CDCA5, CDCA8, CDK1, CENPE, CENPF, CENPW, CKS2, ERCC6L, FAM83D, KIF11, KIF14, KIF2C, KNSTRN, MAD2L1, MASTL, NCAPG, NEK2, OIP5, PRC1, PTTG1, SGO2, SPC25, TPX2, UBE2C
CBX8	GO:0070125	Mitochondrial translational elongation	2.59E−05	MRPL12, MRPL38, MRPL58, MRPS7

*CBXs* chromobox family proteins, *GO* gene ontology.

**Table 3:** Cellular component.

Genes	Significant terms	Description	*p* value	Associated genes
CBX1	GO:0005654	Nucleoplasm	1.97E−05	CBX1, DBF4B, EME1, KAT7, KPNB1, MRPL10, PHB, PNPO, PSMC3IP, SNF8, SRSF1, UBE2Z, UTP18
CBX2	GO:0000775	Chromosome, centromeric region	2.04E−03	CDCA5, CENPW
CBX3	GO:0005925	Focal adhesion	5.69E−07	ENG, FLNC, FLRT2, GSN, LIMS2, LRP1, STARD8, TNS1
CBX4	GO:1990907	Beta-catenin-TCF complex	7.55E−03	TLE4
CBX5	GO:0031618	Nuclear pericentric heterochromatin	1.46E−03	CBX5
CBX6	GO:0031519	PcG protein complex	1.23E−05	CBX6, CBX7
CBX7	GO:0030496	Midbody	3.45E−17	ANLN, ASPM, AURKA, BIRC5, CDCA8, CDK1, CENPE, CENPF, CEP55, ECT2, KIF14, KIF20A, KIF23, KIF4A, NEK2, PLK1, PRC1, RACGAP1
CBX8	GO:0035102	PRC1 complex	3.74E−06	CBX2, CBX4, CBX8

*CBXs* chromobox family proteins, *GO* gene ontology.

**Table 4:** Molecular function.

Genes	Significant terms	Description	*p* value	Associated genes
CBX1	GO:0001850	Complement component C3a binding	1.33E−03	PHB
CBX2	GO:0033678	5′–3′ DNA/RNA helicase activity	1.04E−03	PIF1
CBX3	GO:0034713	Type I transforming growth factor beta receptor binding	1.92E−04	ENG, TGFBR2
CBX4	GO:0051525	NFAT protein binding	3.75E−03	PPARA
CBX5	GO:0008486	Diphosphoinositol-polyphosphate diphosphatase activity	8.67E−04	NUDT4B
CBX6	GO:0004046	Aminoacylase activity	1.39E−03	ABHD14A-ACY1
CBX7	GO:0008017	Microtubule binding	8.43E−09	BIRC5, CENPE, FAM83D, KIF11, KIF14, KIF20A, KIF23, KIF2C, KIF4A, NUSAP1, PLK1, PRC1, RACGAP1
CBX8	GO:0035064	Methylated histone binding	2.48E−04	CBX2, CBX4, CBX8

*CBXs* chromobox family proteins, *GO* gene ontology.

## Discussion

Dysregulation of CBX family proteins affects the development of multiple cancers, including breast cancer. For tumorigenesis and prognosis of breast cancer, despite the identification of the significant functions of some CBX family proteins, the complex and distinct activities of CBXs still require investigation. In this study, we used novel applications of bioinformatics to analyze four aspects of eight CBX proteins in breast cancer: expression pattern, clinicopathological parameters, prognostic value, and genetic alteration.

Human HP1 proteins, HP1α/CBX5, HP1β/CBX1, and HPγ/CBX3, correlated with proliferation, invasion, and metastasis by regulating gene expression in human breast cancer cells^2,17,29^. CBX5 is the most studied HP1 protein, and CBX3 is barely examined. Because of tumor heterogenicity, the expression of HP1 proteins differed in different breast cancer biospecimens. All three HP1 subtypes were positively correlated with the expression level of Ki-67^17^. We found that high CBX1 and CBX3 were associated with poor survival of breast cancer patients. High CBX1 and CBX3 expression was associated with aggressive types of breast cancers (TNBC phenotype), and the patients were more likely to have had lymph node metastasis and P53 mutations. Therefore, CBX1 and CBX3 may function as oncogenes.

CBX5 was upregulated at the mRNA and protein levels in breast cancer cells compared with non-cancerous cells^29,30^. However, CBX5 was downregulated in highly invasive or metastatic breast cancer cell lines compared with weakly invasive or non-metastatic cells, which suggested that CBX5 is a metastatic suppressor in the invasion process^29,31,32^. The suppressor mechanism of CBX5 in invasion is unknown. We also found patients with high CBX5 tended to have less aggressive tumor subtypes (not TNBC phenotype). Prognosis analysis showed that high CBX5 was associated with shorter RFS and DMFS, which suggested that CBX5 functions as an oncogene.

CBX2, CBX4, CBX6, CBX7, and CBX8 are subunits of distinct polycomb repressive 1 complexes that have important functions in the development and progression of breast cancer. CBX2 was overexpressed in breast cancer, and high CBX2 expression was associated with lymph node metastasis, poor tumor differentiation, and high TNM stage^15^. Our results are consistent with those of Zheng et al. who found that CBX2 expression could affect OS and RFS of breast cancer patients independently^15^. Further, we found that patients with high CBX2 tended to have more aggressive tumor subtypes and P53 mutations. Moreover, CBX2 mRNA expression was negatively correlated with CBX7. These results suggested that CBX2 may exert an oncogenic function in breast cancer. Zheng et al. revealed that CBX2 promotes breast tumorigenicity through the PI3K/AKT signaling pathway^15^. The oncogenic mechanism of CBX2 needs further explanation. CBX2 may be an oncogene and a potential therapeutic target for breast cancer. There are no available inhibitors of CBX2. CBX2 contains a chromodomain that binds H3K27me3 with high affinity; this property could be targeted pharmacologically. From the development perspective, a CBX2 antagonist would be a promising therapeutic agent for breast cancer. CBX2 was expressed at low levels in most healthy adult tissues, so CBX2 inhibitors may have few side effects. In addition, CBX2 was overexpressed in breast cancer with poor prognosis, and CBX2 downregulation could inhibit breast tumorigenesis in vivo and vitro. Stage IV patients had higher CBX2 expression compared with other stages, and patients with high CBX2 were more likely to be in positive nodal status and TNBC phenotype. These findings suggested that CBX2 is associated with tumor progression and metastasis.

The mRNA and protein levels of CBX4 were higher in breast cancer tissues than in paired non-cancerous tissues, and high CBX4 expression was independently associated with shorter overall survival^16^. In addition, breast cancer patients with high CBX4 were more likely to have lymph node metastasis and higher clinical stages^16^. CBX4 exerted its oncogenic function through the Notch1 signaling pathway and circular RNA hsa_circ_0008039/miR-515-5p/CBX4 axis^16,33^. However, there is a contradiction between previous studies^16,33^ and our survival measurements. By bioinformatic analysis, patients with high CBX4 had longer OS and RFS, which suggested that CBX4 exerts an anti-cancer effect. Large multicenter prospective studies are required to confirm our results.

Both CBX6 and CBX7 were downregulated in human breast cancer^19,34^. They inhibited breast progression through their pathways. CBX6 controlled a series of genes such as Bone Marrow Stromal cell antigen 2 (BST2) to regulate breast cancer^35–37^. CBX7 repressed breast tumorigenicity by suppressing the Wnt/b-catenin pathway^38^. We found that the most significant difference between breast cancer and normal tissues was the mRNA expression of CBX7. Patients with low CBX6 or CBX7 were more likely to have lymph node metastasis and P53 mutations. These patients tended to have a more aggressive subtype (TNBC phenotype) with poor survival. Besides, CBX6 was positively correlated with CBX7 in breast cancer. These results suggested that CBX6 and CBX7 function as tumor suppressors in breast cancer.

CBX8 functioned in canonical and non-canonical ways to promote breast tumorigenesis^39^. First, polycomb repressive complex 1, the canonical CBX8-containing complex, promoted gene silencing by monoubiquitylation of H2AK119^40^. Second, the non-canonical CBX8 complex, in which CBX8 interacts with Wdr5, promoted the activation of genes in the Notch signaling pathway, regulating normal mammary gland development^39,41^. In addition, CBX8 regulated the p53/p21WAF1 pathway by binding with SIRT1 to suppress premature senescence and growth arrest of breast cancer cells. We found that breast cancer patients with high CBX8 had shorter relapse-free survival compared with low CBX8. These findings suggested that CBX8 is a tumor promoter.

To date, a few investigators have studied the functional significance of CBX proteins in breast cancer. Our investigation consisted of just primarily bioinformatic analyses with some experimental data (i.e., immunohistochemistry). Therefore, extensive prospective clinical studies and other experiments are needed to validate our results. In addition, we need more research that compares CBX proteins with other prognostic markers.

We analyzed the expression, prognostic value, clinicopathological parameters, and Gene Ontology enrichment of eight CBX proteins using several large online databases. We identified the functional significance of these proteins in breast cancer.

## Supplementary information

Supplementary Figure 1A.

Supplementary Figure 1B.

Supplementary Figure 2.

Supplementary Figure 3A.

Supplementary Figure 3B.

Supplementary Table 1.

Supplementary Table 2.

Supplementary Table 3.

Supplementary Table 4.

## Abbreviations

CBXs: Chromobox family proteins
ER: Estrogen receptor
PR: Progesterone receptor
TNBC: Triple-negative breast cancer
SBR: The Scarff–Bloom–Richardson grade
NPI: Nottingham prognostic index
HP1: Heterochromatin protein 1
OS: Overall survival
RFS: Relapse-free survival
PPS: Post-progression survival
DMFS: Distance metastasis-free survival
