# Supplementary figures and images for: Bioinformatic analysis of the expression and prognostic value of chromobox family proteins in human breast cancer
Source: Sci Rep. 2020 Oct 20;10:17739. doi: 10.1038/s41598-020-74792-5 (PMC7576141; doi:10.1038/s41598-020-74792-5)

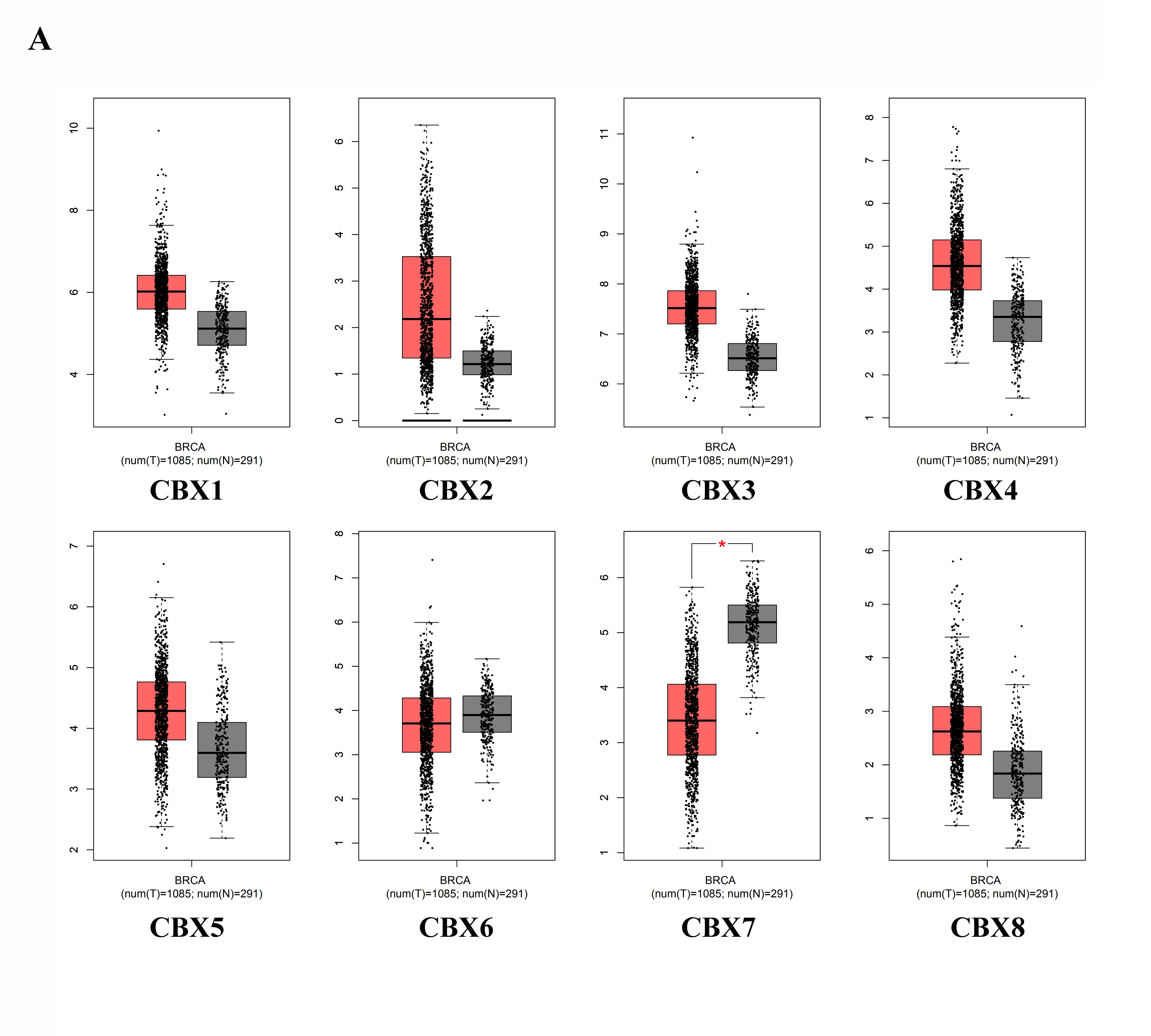

Supplement: Supplementary file 1 — Supplementary Figure 1A. [file 41598_2020_74792_MOESM1_ESM.tif]

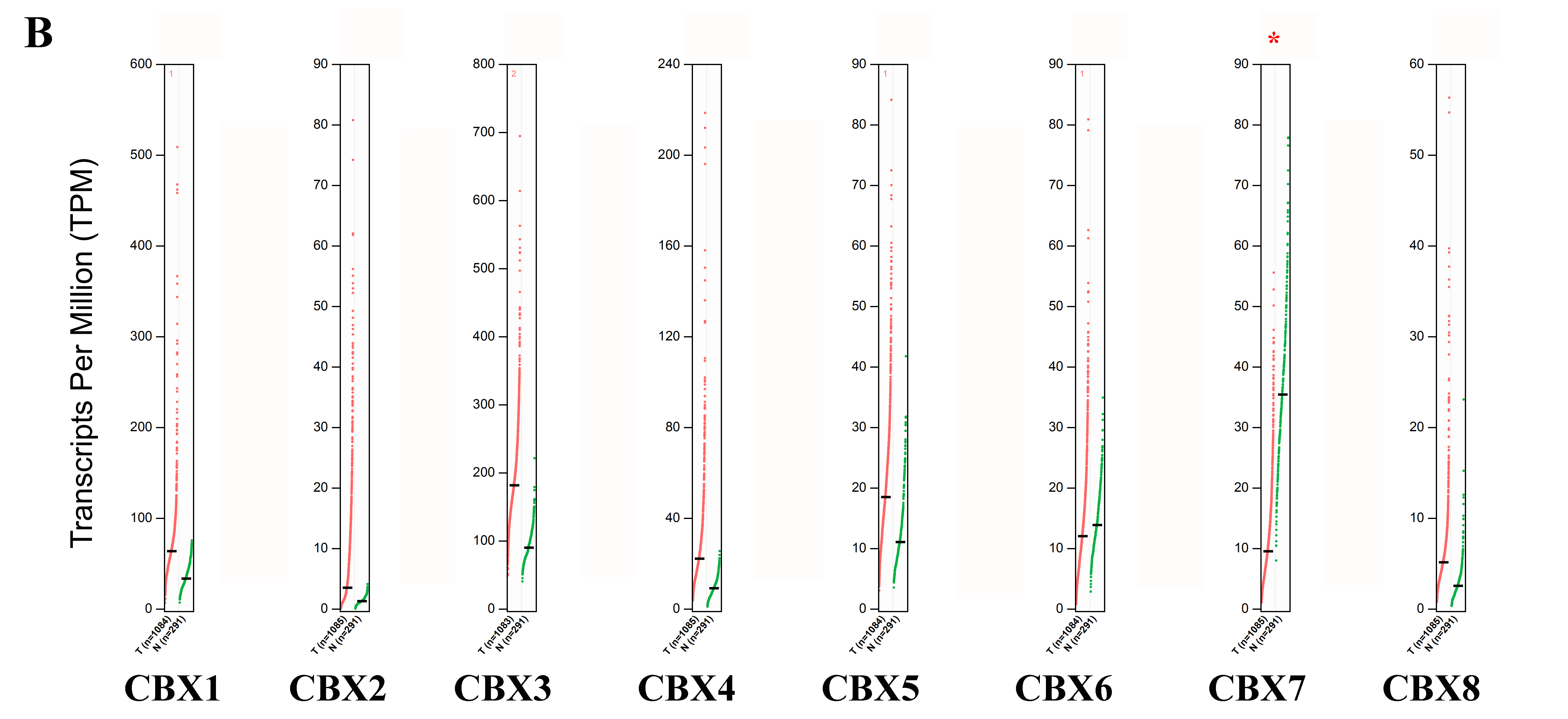

Supplement: Supplementary file 2 — Supplementary Figure 1B. [file 41598_2020_74792_MOESM2_ESM.tif]

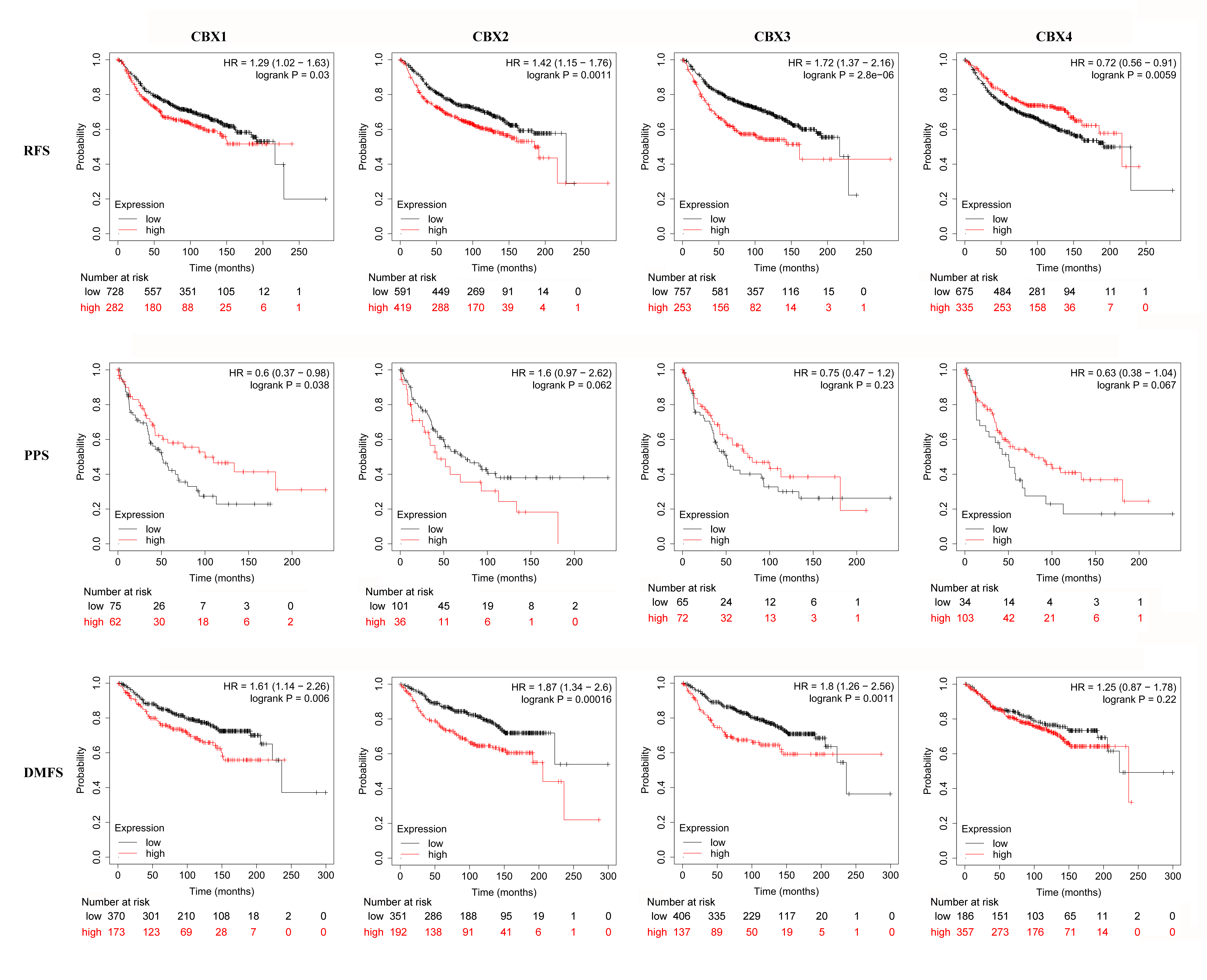

Supplement: Supplementary file 4 — Supplementary Figure 3A. [file 41598_2020_74792_MOESM4_ESM.tif]

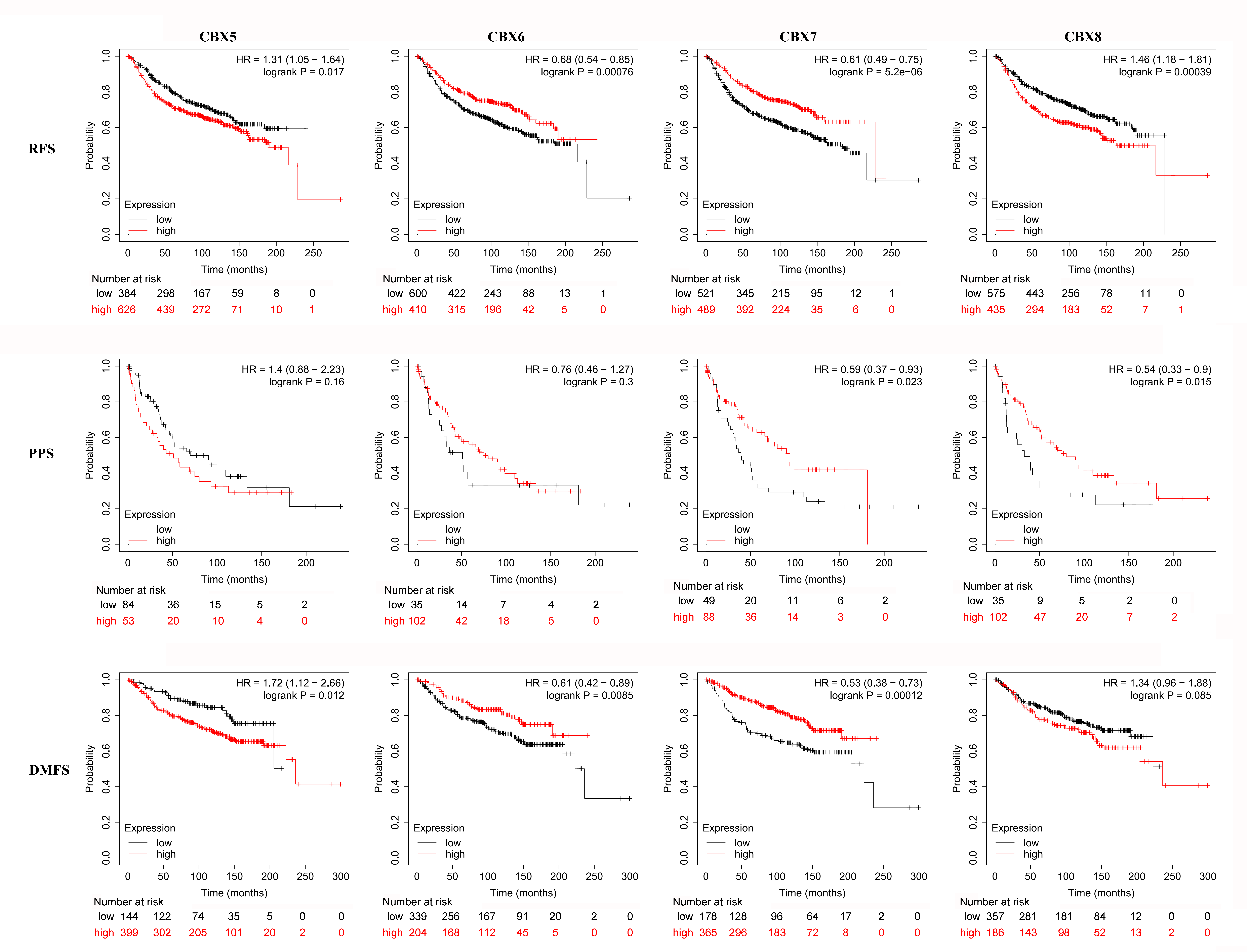

Supplement: Supplementary file 5 — Supplementary Figure 3B. [file 41598_2020_74792_MOESM5_ESM.tif]
