# Supplementary material for: Bioinformatic analysis of the expression and prognostic value of chromobox family proteins in human breast cancer
Source: Sci Rep. 2020 Oct 20;10:17739. doi: 10.1038/s41598-020-74792-5 (PMC7576141; doi:10.1038/s41598-020-74792-5)
Supplement: Supplementary file 6 — Supplementary Table 1. [file 41598_2020_74792_MOESM6_ESM.docx]

| **Supplementary Table 1** Pearson’s correlations (P value) for mRNA expression of pairwise combinations of CBX proteins in cBioPortal and bc-GenExMiner | | | | | | | | |
| --- | --- | --- | --- | --- | --- | --- | --- | --- |
| **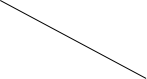** bc-GenExMiner | CBX1 | CBX2 | CBX3 | CBX4 | CBX5 | CBX6 | CBX7 | CBX8 |
|  |  |  |  |  |  |  |  |  |
| cbioportal |  |  |  |  |  |  |  |  |
| CBX1 |  | < 0.0001 | < 0.0001 | < 0.0001 | < 0.0001 | < 0.0001 | < 0.0001 | < 0.0001 |
| CBX2 | 0.267 |  | < 0.0001 | < 0.0001 | < 0.0001 | < 0.0001 | < 0.0001 | < 0.0001 |
| CBX3 | 9.54E-10 | 1.44e-10 |  | < 0.0001 | < 0.0001 | < 0.0001 | < 0.0001 | < 0.0001 |
| CBX4 | 1.69E-03 | 5.18E-13 | 7.47E-04 |  | < 0.0001 | < 0.0001 | 0.4355 | < 0.0001 |
| CBX5 | 5.89E-03 | 4.11E-29 | 7.24E-41 | 0.832 |  | < 0.0001 | < 0.0001 | 0.3491 |
| CBX6 | 4.21E-21 | 3.17E-05 | 0.85 | 0.0306 | 1.08E-05 |  | < 0.0001 | < 0.0001 |
| CBX7 | 0.192 | 3.72E-130 | 1.08E-10 | 0.0268 | 3.10E-44 | 3.34E-93 |  | < 0.0001 |
| CBX8 | 0.746 | 1.17E-64 | 9.53E-15 | 2.87E-107 | 2.43E-12 | 1.19E-05 | 1.12E-21 |  |
